# Evaluation of infrapatellar tendon plication in spastic cerebral palsy with crouch gait pattern: a pilot study

**DOI:** 10.1051/sicotj/2020037

**Published:** 2020-10-08

**Authors:** Mohamed Tageldeen Mohamed, Mohamed Elsobky, Mohamed Hegazy, Hassan M. Elbarbary, Mohamed Mostafa Abdelmohsen, Mostafa Elsherbini, Ahmed Samir Barakat, Nader M. Diab

**Affiliations:** 1 Orthopedics and Traumatology Department, Cairo University Al-Saray Street Manial 11562 Cairo Egypt; 2 Department of Orthopedic Surgery, National Institute of Neuromotor System Corniche Al Nile Street Imbaba Giza Egypt

**Keywords:** Cerebral palsy, Crouch gait, Patella alta, Patellar tendon plication, Pediatric knee, Moment arm

## Abstract

*Objective*: In order to substantially improve crouch pattern in cerebral palsy, the existent patella alta needs to be addressed. This pilot study evaluates the effectiveness of a previously described infrapatellar tendon plication for the treatment of patella alta in crouch gait pattern in skeletally immature spastic cerebral palsy patients. *Methods*: In 10 skeletally immature patients (20 knees) with spastic diplegia and crouch gait, the previously described technique by Joseph et al. for infrapatellar tendon plication was evaluated within the setting of single event multilevel surgery (SEMLS). Outcome measures included knee extension lag, Koshino’s radiological index for patella alta, and the occurrence of complications. Patients were followed-up for a minimum of 12 months. *Results*: The extensor lag improved and was statistically significant in all cases of the study with no incidence of tibial apophyseal injury at the latest follow-up. Radiographic Koshino index normalized and was maintained all through the follow-up period except in one patient (5%) who was overcorrected. Two patients (4 knees, 20%) showed postoperative knee stiffness due to casting which resolved with physiotherapy within six weeks. One knee (5%) developed a superficial infection which also resolved uneventfully with repeated dressings. *Conclusion*: The described infra-patellar plication technique in skeletally immature spastic diplegics appears effective, safe, and reproducible.

## Introduction

The intrigue association between patella alta and crouch knee gait pattern is founded on several factors including quadriceps weakness or spasticity and hamstring spasticity [1]. Persistent flexion all through the stance phase places an excessive workload on the quadriceps muscle, which in turn contracts eccentrically to prevent collapse and to provide sufficient support moment for the limb. This eccentric contraction pulls the patella proximally and may ultimately lead to fragmentation and stress fracture of the patella seen in many cases.

In patella alta there is a decreased moment arm of terminal knee extension since the patella rests on the distal femoral shaft rather than on the contoured femoral condyles. This contributes to a poor moment generation of an already weakened extensor mechanism, resulting in a flexed knee posture and increased tension across the patellofemoral joint which may lead ultimately to degenerative changes in this joint [1].

This pilot study assesses the effectiveness of infrapatellar tendon plication in patella alta of skeletally immature spastic diplegic cerebral palsy (CP) patients by a surgical technique described originally by Joseph and colleagues [2]. Other techniques using tibial tubercle disinsertion and reattachment are technically demanding, potentially risking an apophyseal injury and subsequent growth disturbance, and often require fixation by hardware with subsequent retrieval.

The untainted effect of infrapatellar plication on different gait parameters cannot be completely discerned in these patients undergoing single event multilevel surgery (SEMLS), as the specific biomechanical effect of each procedure or combination of procedures cannot be accurately differentiated. This study therefore primarily focuses on the feasibility and efficacy of infrapatellar plication in the above-described patient group.

## Patients and methods

This pilot study received Research Ethics Committee approval of the Faculty of Medicine, Cairo University, Egypt with the registration number I-500317 and was in full compliance to the Helsinki Declaration and its amendments. This two-center prospective case series was conducted from May 2017 to May 2019 at the National Institute of Neuromotor System, Giza and Abu Elreesh Children University Hospital, Cairo University.

Prior to involvement the parents and legal caregivers were consented after having received detailed information about the procedure, alternatives, and possible complications.

Ten consecutive CP patients (20 knees) were recruited from our outpatient clinics. They encompassed patients below the age of 16 years with spastic diplegia (GMFCS II, III), crouch gait pattern and an extension lag confirmed clinically by extension lag test and patella alta confirmed by Koshino index applied on a plain lateral knee X-rays in 30°–90° knee flexion [3].

Patients with mixed cerebral palsy or dystonia type, with GMFCS I, IV, and V, and patients above the age of 16 years at the time of surgical intervention were excluded from our study. This was to reduce the possible variables and complies with Nahm et al. [4] who reported more consistent and predictable outcomes in spastic CP types.

There were seven male and three female patients, the youngest at the time of surgery was 12 years and the oldest was 15 years old, mean age of patients at the time of surgery was (13.5 years). Four of them were GMFCS II and 6 of them were GMFCS III. All patients underwent infra-patellar tendon plication as a part of a SEMLS which included concomitant soft tissue releases and corrective osteotomies. They consisted of hamstring lengthening, adductor tenotomy, iliopsoas release, gastrocnemius recession, supracondylar femoral extension osteotomy, supracondylar extension derotation osteotomy, supramalleolar derotation tibial osteotomy, and Evan’s osteotomy. Both limbs were operated in the same operative setting by senior surgeons with more than 15 years of experience in pediatric orthopedics (Table 1).

**Table 1:** Demographics and summary of the concomitant surgical procedures.

Total number of patients	*n* = 10
Total number of knees	*n* = 20
Age in years	Mean: 13.5 (range: 12–15)
Gender	7 males and 3 females
GMFCS	4 GMFCS II and 6 GMFCS III
Bony procedures	*n* = 32
Supracondylar extension osteotomy	2
Supracondylar extension derotation osteotomy	13
Supramalleolar derotation osteotomy	8
Evan’s osteotomy	9
Soft tissue procedures	*n* = 25
Iliopsoas tendinous release	6
Adductor tenotomy	6
Hamstring lengthening	8
Gastrocnemius recession	5

**Figure 1:**
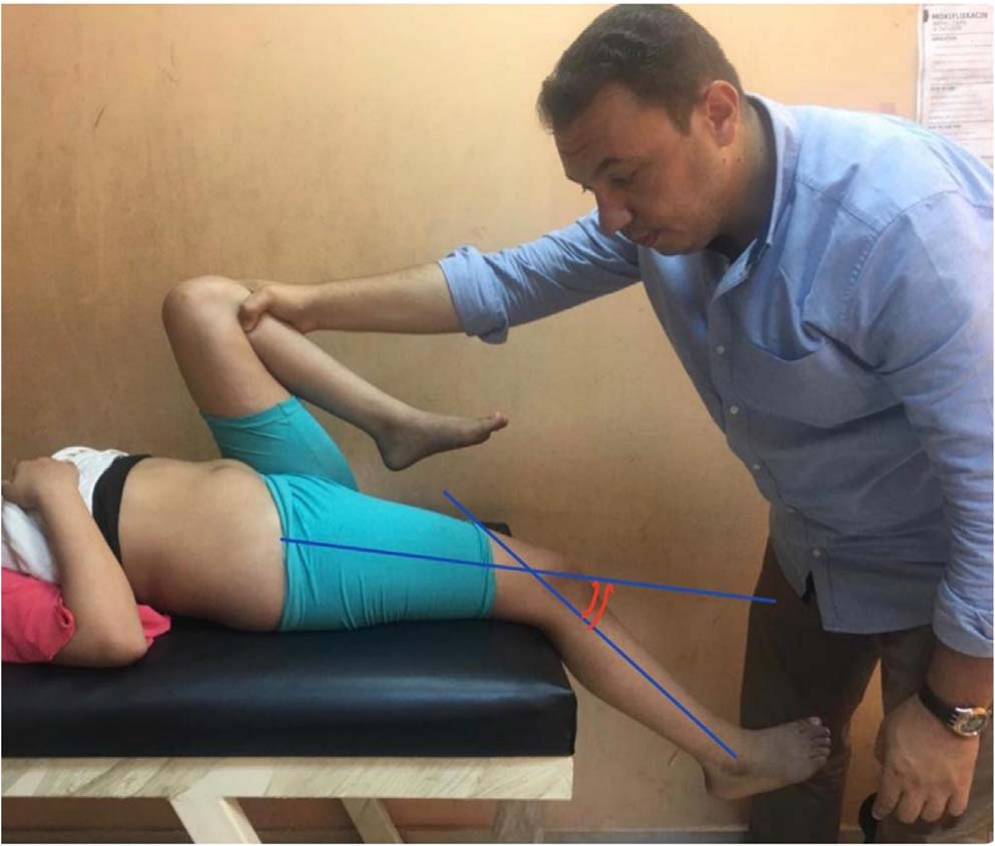
Measurement of the knee extensor lag depicted by the angle at the intersection of both blue lines with ipsilateral hip extension and flexion of the contralateral hip to minimize the stretch of the hamstrings.

Evaluation methods included a detailed history, visual gait observation, general examination, local examination with emphasis on the extensor lag test, and radiographic measurement of the radiological Koshino index in 30°–90° knee flexion. The extensor lag was determined according to Novacheck et al. [5] with the patient supine and the examined hip extended to minimize the stretch of the hamstrings. The latter is further minimized by neutralizing the anterior pelvic tilt effect through over-flexing the contra-lateral hip (Figure 1).

After cast removal, the extensor lag and Koshino index were determined at 3 months and at 12 months follow-up.

## Operative technique

After receiving general and/or caudal anesthesia and a weight-adapted 3rd generation cephalosporin IV short infusion, the patient was placed in the supine position. An above-knee sterile tourniquet was inflated to 250 mmHg after the patient was draped. The sterile tourniquet was deflated after having addressed the knee when iliopsoas recession was necessary in three patients.

A vertical midline anterior knee incision of 8–10 cm was made over the anterior surface of patella and patellar tendon to the level just proximal to the tibial tuberosity. Skin and subcutaneous tissue were dissected, and the anterior surface of patella and patellar tendon was exposed after dividing the paratenon.

The infra-patellar tendon was sharply divided into three equal vertical slips carefully avoiding injury to its insertion at the tibial tuberosity. The two outer slips were sharply dissected from their proximal origin i.e. inferior pole of patella.

Stay sutures were applied to the proximal end of the two outer slips. The knee was fully extended and a curved artery forceps or a dissector was passed under the central slip and used to pull the patella distally to its natural position This position was verified under image intensification in 45° of flexion using the Koshino index and the suture position for the two slips on the anterior surface of the patella was marked. In patients with a dysplastic pole either fragmentation or elongation, we relied on the Blackburne-Peel ratio [6] as it solely relies on the articular patellar rather than the whole patellar length, thus avoiding the fallacies encountered in dysplastic patellae.

Two additional stay sutures were applied on the anterior periosteum of the patella in the previously identified location. The central slip was down pulled followed by tight knotting of both stay sutures on each side which were subsequently reinforced by nonabsorbable 2–0 sutures to securely fix each patellar tendon slips to their new position.

**Figure 2:**
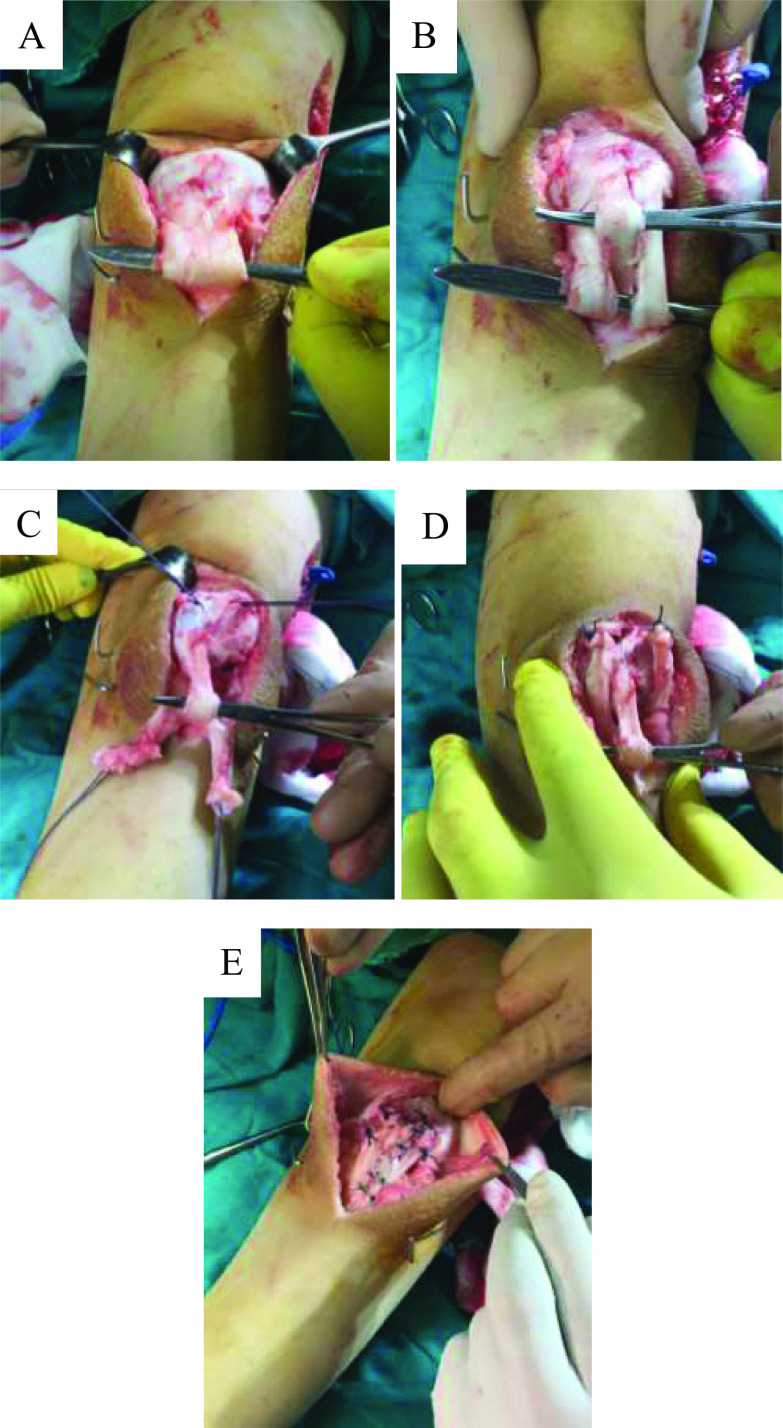
Surgical technique. (A) After midline incision the patellar tendon after dissection of the paratenon is shown. (B) Three equal slips are created by two vertical incisions taking care not to damage the distal insertion into the tibial tuberosity. (C) Stay sutures are applied to the two peripheral slips and by down pulling of the central slip the patella is brought into the desired position. (D) After radiological confirmation of the proper patellar position the stay sutures are tightened over the patella. (E) The central slip is double breasted, the three slips are joined together and augmentation by nonabsorbable suture material is done at the patella.

The central redundant slit was double breasted and stitched up to the highest attainable on the anterior surface of patella by two or three nonabsorbable sutures (Figure 2).

Next, the three slips of the patellar tendon were sutured together to be joined again as one tendon. The tourniquet was deflated followed by meticulous hemostasis and closure of the wound in two layers. An above knee fiberglass cast was applied for six weeks unless more time was required for concomitant operative interventions done within the SEMLS setting.

## Postoperative care

After obtaining plain X-rays, the patients were typically discharged on the second postoperative day and if feasible walker-assisted weight bearing was encouraged. The follow-up schedule was set at 3, 6, and finally at 12 months postoperatively. The lag extension test and Koshino index were upraised preoperatively, at 3 months and at 12 months.

After cast removal (8 weeks in 8 patients, 6 weeks in 2 patients), gentle range of motion was started and once patients regained their ambulatory ability without casts, patients typically began walking with use of ground reaction orthosis for 6 months while maintaining a customized rehabilitation program.

Statistical analysis of the anonymized data was done with SPSS Version 23.0 (IBM, Armonk, NY, USA). Numerical data were expressed in means and paired sample *t* tests were used to compare these means. Frequency analysis was used for categorical data. The confidence interval and *p*-value were set to 95% and 0.05, correspondingly.

## Results

In this pilot study, we relied only on two parameters to assess efficiency and reproducibility of the above -described technique by clinical assessment of knee extension lag and radiographic Koshino index. Other parameters as kinematic gait analysis or video gait analysis and a larger randomized controlled study design clearly would have increased the impact of this study. Hence, this work qualifies as a pilot study to preliminarily determine the feasibility, efficacy, and limitations of the described technique.

Average operative time for the whole procedure was 127 min (range: 80–170 min) and the mean estimated blood loss 56 mL (range: 10–90 mL). The mean follow-up was 16 months and ranged between 12 months to 20 months. The latter two were the overall values for the SEMLS as the infrapatellar plication can never constitute a stand-alone procedure in crouch gait. Nevertheless, these values were not markedly affected by infrapatellar plication.

Statistically significant improvement of the extensor lag could be verified in all patients. Its preoperative mean of 19.85° (range: 10°–46°) decreased to 5.45° (range: 0°–20°) and to 5.95° (range: 0°–19°) at 3 and 12 months after the index operation, respectively.

**Figure 3:**
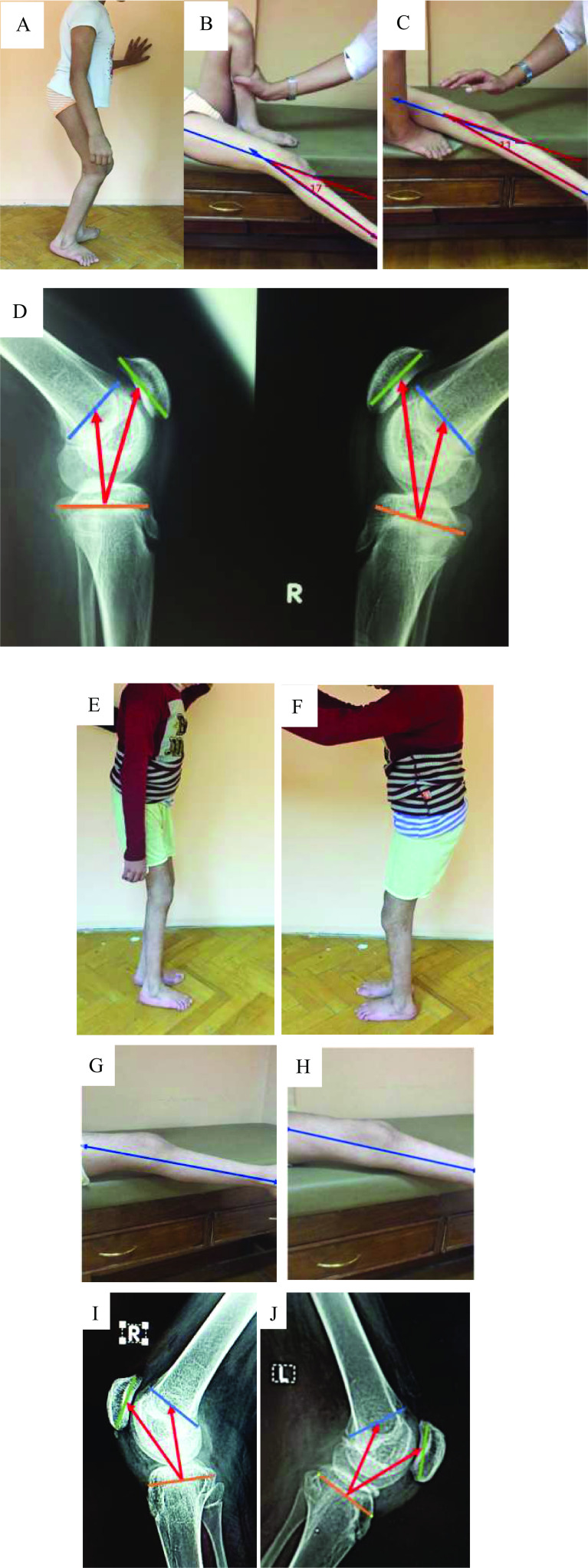
(A) Preoperative clinical presentation of a 13-year-old female with spastic diplegia and crouching gait, GMFCS II. (B) and (C) right and left extension lag of 17° and 11°, respectively. (D) Preoperative Koshino index left 1.5 and right 1.36. (E) and (F) Clinical picture 6 months after bilateral patellar tendon plication, bilateral iliopsoas tendinous release, left rectus femoris intramuscular lengthening and bilateral double column osteotomy. G. and H. Extension lag 5° right and 0° left knee. (I) and (J) Koshino index right knee 1 and left knee 0.9.

The preoperative Koshino index averaged 1.4 (range: 1.30–1.48) and an analogous improvement was noticed at 3 months and 12 months to 1.00 (range: 0.83–1.14) and 1.01 (range: 0.84–1.13), respectively. Both improvements in Koshino index and extension lag were statistically significant and the *p*-value for both were below <.001 (Figure 3).

Paired *t*-testing revealed that the achieved correction of both the extensor lag and the Koshino index was maintained between the 3rd and 12th postoperative months (*p* = .056 and *p* = .171) (Tables 2 and 3).

**Table 2:** Koshino index and extensor lag.

	Koshino index preop.	Koshino index 3 months	Koshino index 12 months	Extension lag preop.	Extension lag 3 months	Extension lag 12 months
*N*						
Valid	20	20	20	20	20	20
Missing	0	0	0	0	0	0
Mean	1.4005	1.0065	1.0115	19.85	5.45	5.95
Standard error of mean	0.01213	0.02000	02069	1.916	1.243	1.311
Median	1.4000	1.0400	1.0300	19.00	5.00	5.00
Standard deviation	0.05424	0.08946	0.09252	8.567	5.558	5.862
Range	0.18	0.31	0.31	36	20	19
Minimum	1.30	0.83	0.84	10	0	0
Maximum	1.48	1.14	1.15	46	20	19

**Table 3:** Paired *T*-test reveals no significant change between the 3rd and 12th months regarding Koshino index and extensor lag.

		Paired differences							
		Mean	Standard deviation	Standard error mean	95% confidence interval of the difference		*t*	*df*	Sig. (2-tailed)
					Lower	Upper			
Pair 1	Koshino index preop. – Koshino Index 3 months	0.39400	0.08804	0.01969	0.35279	0.43521	20.013	19	.000
Pair 2	Koshino Index 3 months – Koshino index 12 months	−0.00500	0.01100	0.00246	−0.01015	0.00015	−2.032	19	.056
Pair 3	Koshino index preop. – Koshino index 12 months	0.38900	0.08944	0.02000	0.34714	0.43086	19.451	19	.000
Pair 4	Extension lag preop. – Extension lag 3 months	14.400	7.500	1.677	10.890	17.910	8.586	19	.000
Pair 5	Extension lag preop. – Extension lag 12 months	13.900	7.615	1.703	10.336	17.464	8.163	19	.000
Pair 6	Extension lag 3 months – Extension lag 12 months	−0.500	1.573	0.352	−1.236	0.236	−1.422	19	.171

Overall complications for patellar tendon plication surgery and associated surgeries amounted to 35%. The most common complication encountered was knee stiffness encountered in two patients (4 knees; 20%). Both patients showed clinical improvement by six weeks after cast removal with adapted physiotherapy. One knee (5%) was overcorrected (Koshino index = 0.84) and required quadriceps muscle exercises, stretching, and improved after 3 months of follow-up. 11 knees out of the 15 knees who underwent supracondylar bony procedures were able to attain squatting and kneeling within 6–12 months after surgery. Bony osteotomies work by reorienting the arc of motion toward the desired direction, they do not, however, increase the total arc in the referred plane. In these patients, the achieved flexion range at the last follow-up was dependent on how successful the reconstruction of the distal femoral anatomy was completed.

Additionally, there was a superficial skin infection in one knee (5%) which resolved with repeated dressings within one week. Another knee (5%) required a surgical revision for femoral derotation due to initial under-correction. During the follow-up period none of our patients showed recurrence of patella alta, patella fracture, or tibial apophyseal injury.

## Discussion

Crouch gait due to extensor mechanism weakness is a relentlessly disabling gait pattern that results in excessive quadriceps load all through stance and slow gait as the step length shortens. In crouch gait, the knee advances faster than the trunk, as the ankle plantar flexors are weak, leading to exaggerated anterior tibial advancement and the quadriceps takes on the load of extending the knee almost all through stance, this excessive load pulls the patella up and this weakens the knee extensor mechanism compromising its ability to hold the knee in near extension. Patella alta and elongation of the patellar tendon develop over time and pose a serious problem in patients with crouch gait.

Joseph et al described this technique in their published study without demonstrating its effectiveness on patients with reproducible data [2]. In this study, we implemented their technique and relied on two parameters, one clinical and the other radiological to assess the effectiveness of their published technique with a follow-up period of one year.

In our series, clinically all patients showed improved extensor lag test results and the patellar position improved in all knees per Koshino index measurement regardless of the concomitant procedures performed (distal femoral extension derotation osteotomy, distal femoral extension osteotomy, hamstring tendons lengthening or another previously mentioned concomitant procedures) which is in agreement with other studies [7–9].

Stout et al. [7] reported the importance of patellar tendon advancement to achieve optimal results in the surgical management of a persistent crouch gait. Their study included 73 patients divided into three groups: (1) a distal femoral extension osteotomy in combination with a distal patellar tendon advancement (33 patients); (2) a distal femoral extension osteotomy without patellar tendon advancement (16 patients); and (3) a distal patellar tendon advancement only (24 patients), all of the study population showed improvement in Koshino index but with a higher rate of over correction compared to our study. Their study reported a complication rate of about 25% which is close to the results of the underlying study. Their technique is technically demanding and in skeletally immature patients, it carried the risk of tibial tubercle apophysis injury as it requires detachment of the patellar tendon and reinsertion into a periosteal sleeve. The above-described techniques are apparently easier and safer as the medial slit remains is not compromised.

In contrast to this study, however, Stout et al. utilized knee kinematic measurements by gait analysis to assess improvement.

Das et al. [8] reported on fourteen adolescents with crouch gait which were treated by supracondylar femoral extension osteotomy (SCFEO) and patellar tendon advancement (PTA) with a follow-up of 3 years. They reported improved knee extension, quadriceps muscle strength, and radiological results similar to our study results. Nevertheless, their technique consisted of complete detachment of the patellar tendon and its reinsertion and hence carried the risk of tibial tubercle apophysis injury.

Sossai et al. [9] reported the outcome of three different approaches for the management of flexed knee gait patients with spastic diplegia. The three surgical procedures were patellar tendon shortening (PTS), PTS combined with rotational osteotomies of the femur and/or tibia, and PTS combined with supracondylar extension osteotomy (SEO) of the distal femur. Their study comprised 24 patients (16 male and 8 female), with a mean age of 16.1 years and a mean of 22 months of follow-up period. Improvement in the mean Koshino index from 1.34 preoperatively to 1.10 postoperatively was reported in the three surgical groups although this study used knee kinematic measurements during gait rather than extension lag test. Their technique carried a risk of tibial tubercle apophysis injury and is technically more demanding as it involved splitting of patellar tendon in the sagittal plane and disinsertion from its distal attachment.

Nevertheless, Hösl et al. [10] reported that the underlying patholgy is not completely reproduced by radiological measures for patella alta. They claim that the responsible radiodiagnostic features are more to be found in the patellar tilt, lengthened patellar tendon, and reduced moment arm.

Our study was limited by the lack of a postoperative kinematic assessment, the short duration of follow-up (12 months) and the small number of patients thus weakening its statistical validity. Furthermore, the isolated effect of this patellar plication on gait improvement is hard to determine since numerous obligatory soft tissue and bony procedures are concomitantly done. Sharan [11] reported that bony procedures can be started at the age of 8 years in CP patients, nevertheless our youngest patient was 12 years old.

Larger comparative multi-centric randomized studies are undoubtedly needed to verify our findings. In the near future, we are hoping for a randomized control study with a bigger sample size, longer duration of follow-up, and spatiotemporal and kinematic gait analysis.

## Conclusion

The described infra-patellar plication technique in skeletally immature spastic diplegics appears effective, safe, and reproducible.

## Conflict of interest

The authors state that there is no conflict of interest and that they did not receive any private or public funding regarding this work, whatsoever.

## Funding

The authors declare that they did not receive any public or private funding, whatsoever.
